# Effects of green tea consumption on glycemic control: a systematic review and meta-analysis of randomized controlled trials

**DOI:** 10.1186/s12986-020-00469-5

**Published:** 2020-07-10

**Authors:** Renfan Xu, Yang Bai, Ke Yang, Guangzhi Chen

**Affiliations:** 1 Department of Medical Ultrasound, Tongji Hospital, Tongji Medical College, Huazhong University of Science and Technology, Wuhan, 430030 P.R. China; 2 Division of Cardiology, Department of Internal Medicine, Tongji Hospital, Tongji Medical College, Huazhong University of Science and Technology, Wuhan, 430030 P.R. China

**Keywords:** Green tea, Catechin, Glycemic control, Meta-analysis

## Abstract

**Background:**

The results of human clinical trials investigating the effects of green tea on glycemic control are inconsistent.

**Methods:**

We conducted a systematic review and meta-analysis of RCTs that examined the effects of green tea supplementation on glycemic control. A literature search in PubMed, Embase, and Cochrane Library databases for RCTs that investigated the effect of green tea consumption on glycemic control was performed up to February 2020. A random-effects model was used to estimate weighted mean difference (WMD) with 95% confidence intervals (CIs).

**Results:**

Twenty-seven trials involving 2194 subjects were included in the meta-analysis. The pooled results showed that green tea significantly lowered fasting blood glucose by − 1.44 mg/dL (95%CI:-2.26, − 0.62 mg/dL; *P* < 0.001) with no obvious heterogeneity (*I*^2^ = 7.7%). However, green tea consumption did not significantly affect fasting insulin and HbA1c values. The mean differences were − 0.46μIU/mL (95% CI: − 1.10, 0.17*μ*IU/mL; *P* = 0.21) for fasting insulin and − 0.06%; (95% CI: − 0.12, 0.01%; *P* = 0.07) for HbA_1c_ concentrations. Heterogeneity was significant in fasting insulin (*I*^2^ = 46.8%) and mild in HbA_1c_ (*I*^2^ = 1.7%).

**Conclusions:**

In short-term trials, green tea supplementation significantly reduced fasting glucose, but had no significant effect on fasting insulin and HbA_1c_. Long-term trials assessing the effects of green tea supplementation on glycemic control are needed.

## Introduction

Type 2 diabetes mellitus (T2DM) is a significant global public health challenge [1]. By the end of 2017, more than 451 million people were living with T2DM worldwide. Moreover, this number is projected to rise to 693 million by 2045 [2]. T2DM is one of the leading risk factors for premature mortality [3]. In addition, T2DM is associated with adverse health outcomes including heart attack, stroke, blindness, kidney failure, and amputation [4]. In prediabetic individuals with impaired glucose tolerance or fasting glucose, lifestyle modification can decelerate the progression to T2DM [3] . However, it is difficult to achieve and sustain sufficient lifestyle intervention. Although medications may play a role in delaying the onset of diabetes, long-term usage may be costly and associated with various side effects. Plants have always been an important source of drugs, and many currently available drugs were either directly or indirectly derived from plants [5]. Herbal drugs are widely used for their effectiveness, relatively low cost, and fewer side effects.

Green tea is produced from the fresh leaves of *Camellia sinensis* and has played an important dietary and medicinal role throughout history, particularly in Asian countries. Green tea contains a variety of effective compounds including antioxidants, vitamins, carbohydrates, protein, minerals, and flavonoid-like polyphenols [6], which may be beneficial in the prevention of diabetes. The most prominent effects of green tea on human health are mainly attributed to catechins, which belong to the flavonoid-like polyphenols family. The four major catechins found in green tea extract are epicatechin (EC), epigallocatechin (EGC), epicatechingallate (ECG), and epigallocatechingallate (EGCG) [7].

A previous meta-analysis with 17 randomized controlled trials (RCTs) suggested that green tea consumption resulted in a significant reduction in fasting blood glucose (FBG) and glycated hemoglobin (HbA_1c_) [8]. In addition, a meta-analysis with 9 cohort studies showed that green tea consumption was associated with a significant reduction of T2DM risk [9]. Both in vitro and animal experiments have shown that green tea catechins, especially EGCG can significant improve glycemic control as well as insulin sensitivity and may lower insulin requirement [10, 11]. However, findings from randomized controlled trials (RCTs) on green tea supplementation and glycemic control or insulin sensitivity in individuals with preclinical diabetes or T2DM are debatable. Some studies observed that green tea significantly improve glycemic control [12], whereas others found no significant association between green tea intake and glycemic control [13, 14].

Given the inconsistency of prior clinical studies and the limitations in these previous meta-analyses, such as the inadequate consideration of possible confounding factors and lower number of included references, we performed a systematic review and meta-analysis to update the evidence which quantitatively assess the effect of green tea supplementation on measures of glucose control and insulin sensitivity.

## Methods

### Search strategy and eligibility criteria

This systematic review and meta-analysis was conducted in accordance with the recommendations outlined in the PRISMA (Preferred Reporting Items for Systematic Reviews and Meta-Analyses) statement [15]. Relevant English-language articles were identified via searches in PubMed, Embase, and the Cochrane Library from the index date of each database through February 2020. The search terms were as follows: “green tea,” “green tea extract,” “tea component(s),” “tea solid(s),” “tea polyphenols,” “catechin,” “catechins,” “EGCG,” and “*Camellia sinensis*,” which were paired with the following words: “glucose,” “glyc(a)emia,” “hyperglycaemia,” “glucose control,” “insulin,” “insulin sensitivity,” “insulin resistance,” “HbA_1c_,” “glycated protein,” “fructosamine,” and “diabetes”. Additional studies were identified by manually screening the reference of originally identified reviews and research reports or the clinical trials. The search was confined to studies involving humans.

### Study selection

The prespecified inclusion criteria were as follows: 1) RCTs with both parallel and crossover interventions, 2) study involved adult subjects who consumed green tea for≥2 weeks, 3) blood glucose was evaluated by estimating the concentrations of FBG, fasting blood insulin (FBI) and HbA_1c_, and 4) the study used a concurrent control group with the only difference between the treatment and control groups being the consumption of either green tea or green tea extract. The exclusion criteria were as follows: 1) subjects in each group ≤10, 2) green tea extract was given as part of a multicomponent supplement,3) RCTs that did not report mean (SD) changes in fasting glucose, fasting insulin, or HbA_1c_ in each treatment group and could not be calculated from the data available. The data from multiple published reports involving the same study population were included only once.

### Quality assessment

Two review authors (CGZ and XRF) independently assessed the study quality and any disagreement was resolved by discussion between the third author (YK). Jadad scoring criteria was used in which a study was judged on 0–5 points (5 reflected the highest quality). With this system, one point was allocated to each for 1) randomization; 2) double blinding (participant and researcher masking); 3) reporting the number of and reasons for withdrawal; 4) generation of random numbers; and 5) allocation concealment. Trials were considered of high-quality if the Jadad score was ≥4, while trials were classified as low-quality if the Jadad score was < 4 [16].

### Data extraction

Two authors (CGZ and XRF) independently extracted the data, and any discrepancies between the two reviewers were resolved through discussion with a third author (BY). The following information was recorded using a standardized electronic form: study characteristics (the first author, publication year, study design, study duration, sample size, intervention type, and dosage), population information (age, sex, country, and baseline fasting glucose), and baseline and final concentrations or net changes of FBG, FBI, HbA1c and homeostatic model assessment of insulin resistance (HOMA-IR). Studies with multiple dosages of green tea or multiple control groups were included separately in the meta-analysis.

### Statistical analysis

A meta-analysis was performed with the use of the STATA statistical software (version 11; STATA Corp LP). For parallel trials, the treatment effects were calculated as the weighted mean difference (WMD) and standard deviation (SD) in the change from baseline to follow-up in the green tea group versus control group. For crossover trials, the treatment effects were calculated as the WMD and SD at follow-up in the green tea intervention versus control periods. If the SD were not reported directly, the variances were imputed from 95% CIs, *P* values, standard error (SE), or *t* values [17]. In addition, missing SD values for paired differences were imputed by assuming a correlation coefficient of 0.5 between variances at baselines and completion of trials according to the method of Follmann et al. [18]. The statistical heterogeneity of treatment effects between studies was evaluated via the Cochran’s Q test (*P* < 0.1 was considered significant) and the inconsistency index (*I*^2^). *I*^2^ > 50% indicated significant heterogeneity across studies [19]. Random-effects models (DerSimonian and Laird), which considered both within- and between-study variation, were performed for the studies used different doses, different populations, different durations and so on [20]. Primary outcome measures included WMD in FBG, FBI, and HbA_1c_ after green tea supplementation. The secondary outcome measures included WMD in HOMA-IR concentration.

Sensitivity analyses were used to evaluate the stability of the results by removing a single study each time to identify the effect of individual studies on the pooled effect size. Prespecified subgroup analyses were performed by catechins dosage (≥500 mg/d compared with < 500 mg/d), intervention type (green tea beverage compared with green tea capsule), participants’ country (Asian compared with Western countries), study design (parallel compared with crossover), baseline fasting glucose level (high or normal), trial quality (low risk of bias, some concern or high risk of bias) and treatment duration (≥12 weeks compared with < 12 weeks). In addition, the study duration< 12 weeks were defined as short duration for the changes of HbA_1c_ need to be monitored for at least 2–3 months. Meta-regression analysis was performed to examine the association between the net change in fasting glucose, fasting insulin or HbA_1c_ and intervention dose, treatment duration, intervention type, caffeine content, different ethnicity or study design. Publication bias was assessed by funnel plots and Egger’s test [21]. A *P* value of < 0.05 was considered statistically significant, unless otherwise specified.

## Results

### Results of the literature search

The search strategy identified 2324 abstracts. After the titles and abstracts were screened, 2229 articles were excluded and 95 articles underwent full-text review. A further 68 articles were excluded for the following reasons: 26 articles did not provide relevant outcomes, 14 articles involved green tea as a multicomponent supplement in the experimental group, 12 articles were excluded because the subjects had been treated with black tea or oolong tea.

**Fig. 1:**
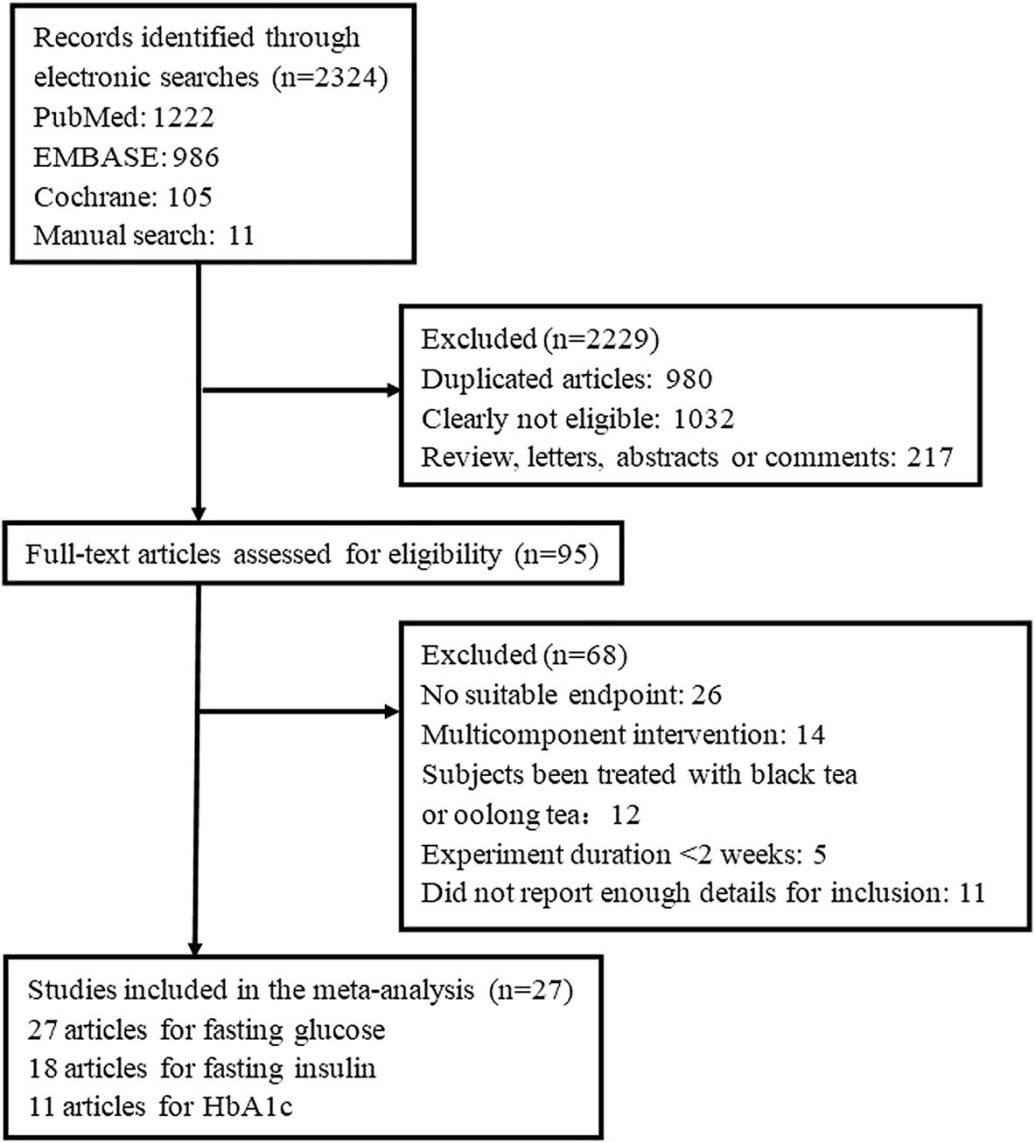
Flow diagram of the trial selection process

5 studies were less than 2 weeks in duration and 11 articles did not report sufficient details for inclusion. Finally, 27 eligible articles met the inclusion criteria and were included in the meta-analysis (Fig. 1).

### Study characteristics

Twenty seven eligible RCTs [22–48] were enrolled in this meta-analysis (Table 1). Twenty seven studies [22–48] with 2194 subjects reported on FBG, 18 studies [23–27, 29, 31–37, 39, 43, 44, 48] with 1559 subjects reported data on FBI and 11 studies [22, 24, 27, 31, 32, 34, 37, 40, 41, 43, 48] with 767 subjects reported data on HbA_1c_. The green tea catechins intake ranged from 80 to 1344 mg/d, the trial size varied from 25 to 240 subjects and the study duration ranged from 3 weeks to12 months. (Table 1).

**Table 1:** Characteristics of 27 included randomized controlled trials

Reference	Study design	No. of subjects (M/F)	Country or Region	Age(y)^a^	BMI (kg/m^2^)^a^	FBG (mmol/) (GT/C)	FBI (μIU/ml) (GT/C)	HbA1c (%) (GT/C)	Duration	Tea group	Control group	Type of diet
Basu 2011 [22]	P	25(5/20)	USA	42.5 ± 1.7	36.1 ± 1.3	5.0/4.9	NA	5.5/5.6	8wk	DGTE beverage (928 mg catechins)	Placebo (water)	Usual diet
Bogdanski 2012 [23]	P	56(28/28)	Poland	30–60	33.2 ± 2.8	5.5/5.6	32.0/31.8	NA	3mo	GTE capsule (208 mg EGCG)	Placebo (cellulose)	Usual diet
Brown 2009 [24]	P	88(88/0)	UK	40–65	31.2 ± 2.8	5.4/5.3	11.1/10.7	5.3/5.1	8wk	DGTE capsule (800 mg EGCG)	Placebo (lactose)	Usual diet
Brown 2011 [25]	C	66(66/0)	UK	40–69	31.7 ± 2.7	5.9/6.0	12.5/12.1	NA	6wk	DGTE capsule (800 mg catechins)	Placebo (lactose)	Usual diet
Chan 2006 [26]	P	34(0/34)	China	25–40	30.5 ± 1.9	5.1/5.2	7.3/13.8	NA	3mo	GTE capsule (661.3 mg cathchins, 152.8 mg caffeine)	Placebo	Usual diet, caffeine-free
Chen 2016	P	77(0/77)	Taiwan	44.5 ± 11.4	30.5 ± 3.7	5.5/5.9	19.6/16.5	5.8/6.2	12wk	DGTE capsule (1344 mg catechins)	Placebo (cellulose)	Usual diet
Diepvens 2006 [28]	P	46(0/46)	Netherlands	19–57	27.7 ± 1.8	5.2/5.2	NA	NA	12wk	GTE capsule (1125 mg catechins, 225 mg caffeine)	Placebo	Low-energy diet
Dostal 2016	P	237(0/237)	USA	60.7 ± 5.0	28.2 ± 2.9	5.4/5.4	6.7/6.2	NA	12mo	DGTE capsule (1315 mg catechins)	Placebo (maltodextrin and cellulose)	Usual diet with exercise
Frank 2009 [30]	P	33(33/0)	UK	18–55	26.7 ± 3.3	3.9/3.8	NA	NA	3wk	GTE capsule (672 mg catechins, 114 mg caffeine)	Placebo (matched with caffeine)	Usual diet with exercise, limit tea and coffee
Fukino 2005 [31]	P	66(53/13)	Japan	53.5 ± 8.0	25.7 ± 4.3	7.5/7.8	8.7/10.3	6.2/6.1	2mo	GTE beverage (456 mg catechins, 102 mg caffeine)	Not report	Usual diet
Fukino 2008 [32]	C	60(49/11)	Japan	32–73	25.5 ± 4.8	7.5/7.7	8.8/10.3	6.2/6.1	2mo	GTE beverage (456 mg catechins, 102 mg caffeine)	No intervention	Usual diet
Hill 2007 [33]	P	38(0/38)	Australia	45–70	25–39.9	5.4/5.5	11.0/8.1	NA	12wk	DGTE capsule (300 mg EGCG)	Placebo (lactose capsules)	Usual diet with exercise
Hsu 2008 [34]	P	78(0/78)	Taiwan	16–60	> 27	6.3/5.8	16.1/13.1	NA	3mo	GTE capsule (613.5 mg cathchins, 27.3 mg caffeine)	Placebo	Usual diet
Hsu 2011 [35]	P	68(24/44)	Taiwan	20–65	> 25	9.5/9.7	14.5/11.4	8.4/8.4	16wk	DGTE capsule (1344 mg catechins)	Placebo (cellulose)	Usual diet
Kovacs 2004 [36]	P	104(26/78)	Netherlands	18–60	25–35	5.7/5.	11.0/10.3	NA	13w	GTE capsule (573 mg cathchins, 104 mg caffeine)	Placebo	Usual diet
Liu 2014 [37]	P	77(32/45)	Taiwan	54.3 ± 6.8	26.3 ± 4.4	7.7/8.5	15.6/17.0	7.5/7.7	16wk	DGTE capsule (1344 mg catechins)	Placebo (cellulose)	Usual diet
Lu 2016 [38]	P	64(0/64)	Taiwan	29.1 ± 8.9	21.2 ± 4.4	5.0/4.7	NA	NA	4wk	DGTE capsule (1344 mg catechins)	Placebo (cellulose)	Usual diet
Mielgo-Ayuso 2014 [39]	P	83(0/83)	Spain	18–49	34 ± 2.8	5.0/5.1	9.6/8.4	NA	12wk	300 mg EGCG	Placebo (lactose)	Usual diet
Mirzaei 2009 [40]	P	82(16/66)	Iran	54.6 ± 11.2	29.9 ± 4.2	9.0/9.8	15.9/14.1	7.2/7.6	8w	GTE capsule (240 mg polyphenols, 150 mg caffeine)	Placebo (cellulose)	Usual diet
Miyazaki 2013 [41]	P	52(20/32)	Japan	68.7 ± 6.3	22.6 ± 3.0	5.6/5.4	NA	5.5/5.1	14wk	GTE beverage (630.9 mg catechins, 77 mg caffeine)	GTE beverage (88.7 mg catechin, 82.4 mg caffeine)	Low-energy diet,limit exercise
Nagao 2007 [42]	P	240 (140/100)	Japan	25–55	24–30	5.4/5.2	NA	NA	12wk	GTE beverage (582.8 mg catechins, 72.3 caffeine)	GTE beverage (96 mg catechins, matched with caffeine)	Usual diet with exercise
Nagao 2009 [43]	P	43(18/25)	Japan	64.9 ± 7.3	25.6 ± 3.7	7.5/7.2	7.4/6.3	6.7/6.6	12wk	GTE beverage (582.8 mg catechins, 72.3 caffeine)	GTE beverage (96 mg catechins, matched with caffeine)	Usual diet
Ryu 2006 [44]	C	55(31/24)	Korea	53.9 ± 7.7	25 ± 2.2	6.7/6.9	10.3/10.4	NA	4wk	GTE beverage (9 g green tea)	Placebo (water)	Usual diet
Sone 2011 [45]	P	51(18/33)	Japan	20–70	25 ± 4	5.3/5.5	NA	NA	9wk	GTE beverage (400 mg catechins, 105 mg caffeine)	GTE beverage (100mgcatechins, 80 mg caffeine)	Usual diet, limit catechins
Suliburska 2012 [46]	P	46(23/23)	Poland	30–60	32.8 ± 2.5	5.6/5.7	NA	NA	3mo	GTE capsule (208 mg EGCG)	Placebo (cellulose)	Usual diet
Tadayon 2018	P	79(0/79)	Iran	53.3 ± 3.9	29.9 ± 4.1	5.1/5.2	NA	NA	4wk	GTE capsule (80-94 mg polyphenol)	Placebo	Usual diet
Wu-a 2012 [48]	P	69(0/69)	USA	> 45	26.8–31.8	5.5/5.4	8.5/7.3	5.9/5.9	2mo	DGTE capsule (400 mg EGCG)	Placebo	Usual diet
Wu-b 2012 [48]	P	66(0/66)	USA	> 45	26.8–31.4	5.7/5.4	9.5/7.3	6.3/5.9	2mo	DGTE capsule (800 mg EGCG)	Placebo	Usual diet

^a^Data expressed as a mean with standard deviation. FBG, fasting blood glucose; FBI, fasting blood insulin; HbA1c,glycated hemoglobin; GTE, green tea extract; DGTE, decaffeinated green tea extract; EGCG, epigallocatechin gallate; P, parallel trial; C, crossover trial; wk., week; mo, month; M, male; F, female

Of the 27 trials with 28 comparisons included in the current meta-analysis, 13 comparisons [22–25, 28–30, 33, 36, 39, 46, 48] were conducted in western countries and 15 comparisons [26, 27, 31, 32, 34, 35, 37, 38, 40–45, 47] were conducted in Asian countries. Twenty comparisons [22–30, 33, 36, 38, 39, 41, 42, 45–48] were performed in subjects with normal FBG and 8 comparisons [31, 32, 34, 35, 37, 40, 43, 44] were performed in subjects with high level FBG. Most comparisons (25 of 28) used a parallel study design [22–24, 26–31, 33–43, 45–48], while others (3 comparisons) used a crossover design [25, 32, 44]. Twelve comparisons [22, 24, 25, 27, 29, 33, 35, 37–39, 48] adjusted for the confounding effect of caffeine on glucose and insulin, 13 comparisons [26, 28, 30–32, 34, 36, 40–45] used caffeinated green tea, and 3 [23, 46, 47] did not report the use of coffee. Twenty comparisons [23–30, 33–40, 46–48] used green tea extract capsule and eight comparisons [22, 31, 32, 41–45] used green tea beverage (Table 1).

### Data quality

The study quality of the 27 included RCTs varied. Fourteen studies [22–27, 29, 34, 35, 38, 39, 45–47] were classified as high-quality (Jadad score ≥ 4), and the remaining 13 studies [28, 30–33, 36, 37, 40–44, 48] were classified as low-quality (Jadad score < 4). Most trials did not report details regarding allocation concealment (14 of 27) [26, 28, 30–32, 36, 37, 40–44, 46, 48] or randomization method (15 of 27) [23, 28, 30–33, 36, 37, 40–45, 48]. Twenty-two trials used double-blinded design [23–30, 34–43, 45–48], one trial used a single-blinded design [22], and four trials used an open-label design [31–33, 44]. Three trials did not report the dropout rate or the reasons for the dropouts [28, 40, 44] (Table 2).

**Table 2:** Validity of included studies

References	Randomization	Allocation concealment	Masking of participants	Masking of researches	Generation of random numbers reported	Reporting of withdraws	Jadad score
Basu 2011 [22]	Yes	Adequate	Yes	No	Yes	Yes	4
Bogdanski 2012 [23]	Yes	Adequate	Yes	Yes	No	Yes	4
Brown 2009 [24]	Yes	Adequate	Yes	Yes	Yes	Yes	5
Brown 2011 [25]	Yes	Adequate	Yes	Yes	Yes	Yes	5
Chan 2006 [26]	Yes	Unclear	Yes	Yes	Yes	Yes	4
Chen 2016	Yes	Adequate	Yes	Yes	Yes	Yes	5
Diepvens 2006 [28]	Yes	Unclear	Yes	Yes	No	No	2
Dostal 2016	Yes	Adequate	Yes	Yes	Yes	Yes	5
Frank 2009 [30]	Yes	Unclear	Yes	Yes	No	Yes	3
Fukino 2005 [31]	Yes	Unclear	No	No	No	Yes	2
Fukino 2008 [32]	Yes	Unclear	No	No	No	Yes	2
Hill 2007 [33]	Yes	Adequate	No	No	No	Yes	3
Hsu 2008 [34]	Yes	Adequate	Yes	Yes	Yes	Yes	5
Hsu 2011 [35]	Yes	Adequate	Yes	Yes	Yes	Yes	5
Kovacs 2004 [36]	Yes	Unclear	Yes	Yes	No	Yes	3
Liu 2014 [37]	Yes	Unclear	Yes	Yes	No	Yes	3
Lu 2016 [38]	Yes	Adequate	Yes	Yes	Yes	Yes	5
Mielgo-Ayuso 2014 [39]	Yes	Adequate	Yes	Yes	Yes	Yes	5
Mirzaei 2009 [40]	Yes	Unclear	Yes	Yes	No	No	2
Miyazaki 2013 [41]	Yes	Unclear	Yes	Yes	No	Yes	3
Nagao 2007 [42]	Yes	Unclear	Yes	Yes	No	Yes	3
Nagao 2009 [43]	Yes	Unclear	Yes	Yes	No	Yes	3
Ryu 2006 [44]	Yes	Unclear	No	No	No	No	1
Sone 2011 [45]	Yes	Adequate	Yes	Yes	No	Yes	4
Suliburska 2012 [46]	Yes	Unclear	Yes	Yes	Yes	Yes	4
Tadayon 2018	Yes	Adequate	Yes	Yes	Yes	Yes	5
Wu 2012 [48]	Yes	Unclear	Yes	Yes	No	Yes	3

### Main outcomes

**Fig. 2:**
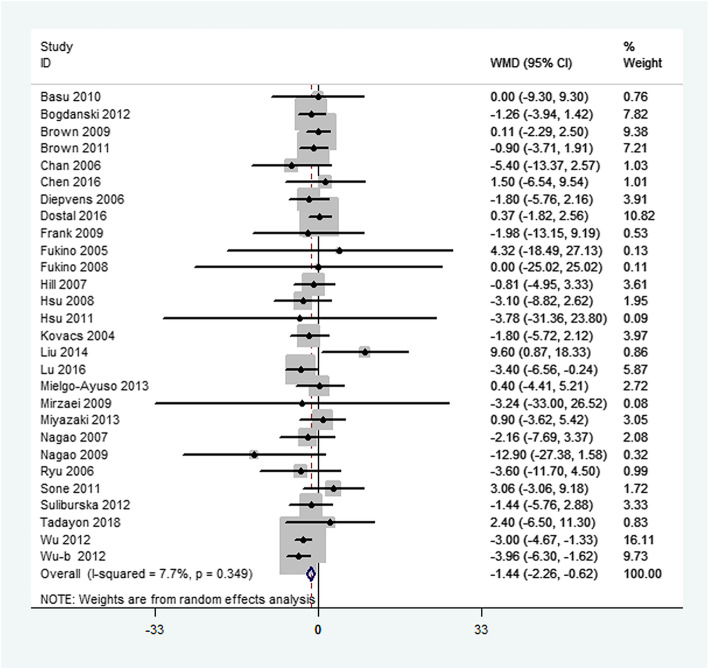
Meta-analysis of the effects of green tea on fasting blood glucose concentrations. Results from individual trials were pooled with the use of random-effect models and are expressed as weighted mean differences with 95% CIs

**Fig. 3:**
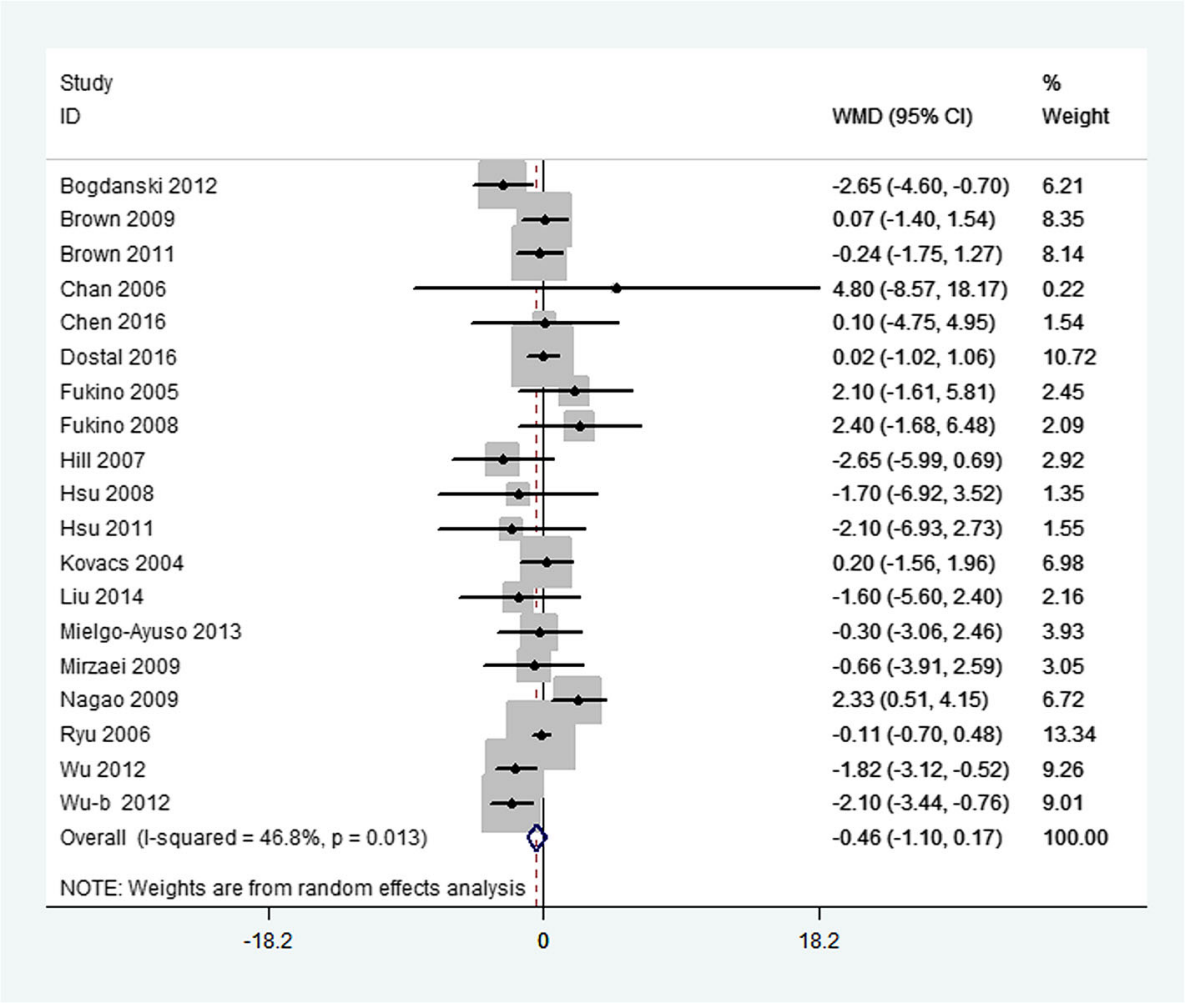
Meta-analysis of the effects of green tea on fasting blood insulin concentrations. Results from individual trials were pooled with the use of random-effect models and are expressed as weighted mean differences with 95% CIs

**Fig. 4:**
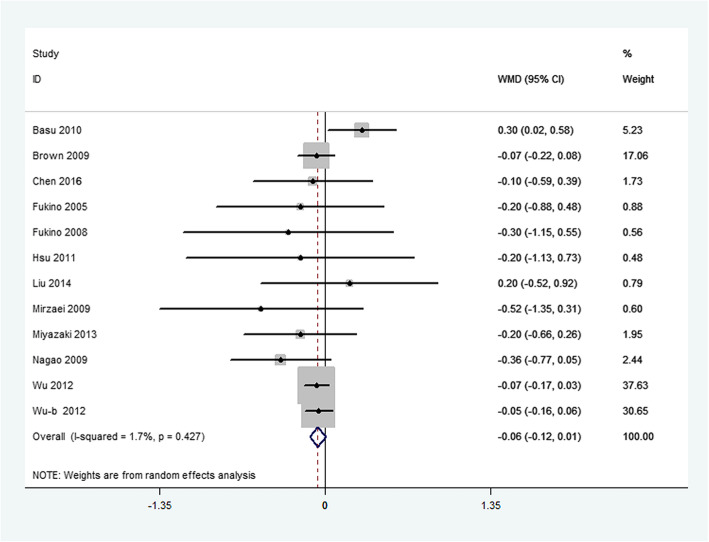
Meta-analysis of the effects of green tea on HbA_lc_ concentrations. Results from individual trials were pooled with the use of random-effect models and are expressed as weighted mean differences with 95% CIs

Primary outcome measures included changes in FBG, FBI, and HbA_1c_. Overall, green tea supplementation significantly decreased FBG concentration by − 1.44 mg/dL (95%CI:-2.26, − 0.62 mg/dL; *P* < 0.001). Heterogeneity was not significant for this outcome (*I*^2^ = 7.7%, *P* = 0.35) (Fig. 2). Green tea supplementation had no significant effect on FBI concentrations in the overall analysis (WMD: − 0.46μIU/mL; 95%CI: − 1.10, 0.17*μ*IU/mL; *P* = 0.21). The overall test for heterogeneity was significant (*I*^2^ = 46.8%; *P* = 0.01) (Fig. 3). In addition, there was no significant difference in serum HbA_1c_ concentration between green tea supplementation and placebo group (WMD: − 0.06%; 95% CI: − 0.12, 0.01%; *P* = 0.07), with mild heterogeneity (*I*^2^ = 1.7%; *P* = 0.43) (Fig. 4).

Secondary outcome measures included changes in HOMA-IR concentration. Green tea supplementation had no significant effect on HOMA-IR (WMD: -0.15; 95%CI:-0.39, 0.10; *P* = 0.24) compared with controls. Heterogeneity was not significant for this outcome (*I*^2^ = 34%, *P* = 0.12).

### Subgroup analysis and meta-regression

In the subgroup analysis, green tea consumption significantly lowered FBG concentrations in subjects using green tea capsule or with high catechins dosage, subjects from western countries, subjects in short duration of green tea supplementation, subjects with normal FBG, studies with caffeinated green tea intake, studies with parallel design, and studies with low quality. However, significant reduction in fasting glucose was not found in other subgroups. In addition, the beneficial effect for green tea supplementation on fasting insulin was observed in subjects with green tea capsule, subjects from western countries, subjects with normal baseline FBG and studies with decaffeinated green tea intake. However, no effect was found in other subgroups. Significant reductions in HbA_1c_ concentrations were observed in subjects from Asian countries, studies with caffeine in green tea and studies with low quality, while the obvious effect was not found in other subgroups (Table 3).

**Table 3:** Subgroup analyses of fasting blood glucose, fasting blood insulin and HbA1c stratified by previously defined study characteristics

	Change in FBG			Change in FBI			Change in HbA1c		
Subgroup	Trials (n)	Net change (95%CI) (mg/dl)	I^2^	Trials (n)	Net change (95%CI) (mg/dl)	I^2^	Trials (n)	Net change (95%CI) (mg/dl)	I^2^
Type of intervention									
Green tea beverage	8	−0.37(−3.00, 2.27)	0	4	1.28(− 0.48, 3.03)	64	5	−0.10(− 0.42, 0.22)	55
Green tea capsule	20	−1.63(−2.60,-0.66)	22	15	− 0.88(− 1.54, − 0.22)	29	7	− 0.06(− 0.13, 0.00)	0
Duration									
≥ 12 weeks	15	− 1.04(− 2.30,0.22)	12	11	− 0.35(− 1.48, 0.78)	47	5	− 0.19(− 0.42, 0.05)	0
< 12 weeks	13	− 2.09(− 3.14, − 1.03)	4	8	− 0.55(− 1.41, 0.30)	56	7	− 0.04(− 0.13, 0.05)	25
Country									
Western	13	− 1.60(− 2.50, − 0.69)	11	9	− 0.88(− 1.71,-0.05)	56	4	− 0.02(− 0.13, 0.08)	52
Asian	15	− 1.20(− 3.30, 0.90)	20	10	0.40(− 0.58, 1.37)	20	8	− 0.22(− 0.42, − 0.01)	0
Catechins dose									
≥ 500 mg/dl	17	−1.68(− 2.93,-0.43)	39	11	− 0.32(− 1.24, 0.60)	58	9	− 0.05(− 0.13, 0.03)	16
< 500 mg/dl	10	− 0.82(− 2.46,0.83)	0	7	− 0.79(− 2.31, 0.74)	36	3	− 0.32(− 0.77, 0.13)	0
Caffeine									
With caffeine	13	−2.00(− 3.78, − 0.22)	0	8	0.72(− 0.29, 1.73)	31	5	− 0.30(− 0.55, − 0.05)	0
Without caffeine	12	−1.25(− 2.65, 0.15)	47	10	−0.89(− 1.59, − 0.19)	27	7	− 0.04(− 0.11, 0.04)	12
Study design									
Parallel	25	−1.51(− 2.49, − 0.53)	21	16	− 0.57(− 1.43, 0.28)	53	11	− 0.06(− 0.13, 0.02)	8
Crossover	3	−1.17(− 3.80, 1.46)	0	3	− 0.08(− 0.63, 0.46)	0	1	−0.30(− 1.15, 0.55)	NA
Fasting blood glucose									
High(> 6.1 mmol/l)	8	−1.16(−6.22, 3.90)	26	8	0.41(−0.75,1.57)	35	6	−0.26(− 0.52,0.01)	0
Normal (3.9–6.1 mmol/l)	20	−1.56(−2.41, − 0.72)	11	11	−0.83(− 1.61,-0.05)	47	6	− 0.04(− 0.12,0.04)	26
Study quality									
High quality	14	−0.77(−1.78,0.24)	0	9	−0.39(−1.04,0.27)	0	4	0.03(−0.20, 0.27)	47
Low quality	14	−2.44(−3.74,-1.15)	10	10	−0.27(−1.33, 0.79)	67	8	−0.08(− 0.15, − 0.01)	0

*FBG* fasting blood glucose, *FBI* fasting blood insulin, *HbA*_*1c*_ glycated hemoglobin, *NA* not applicable

**Fig. 5:**
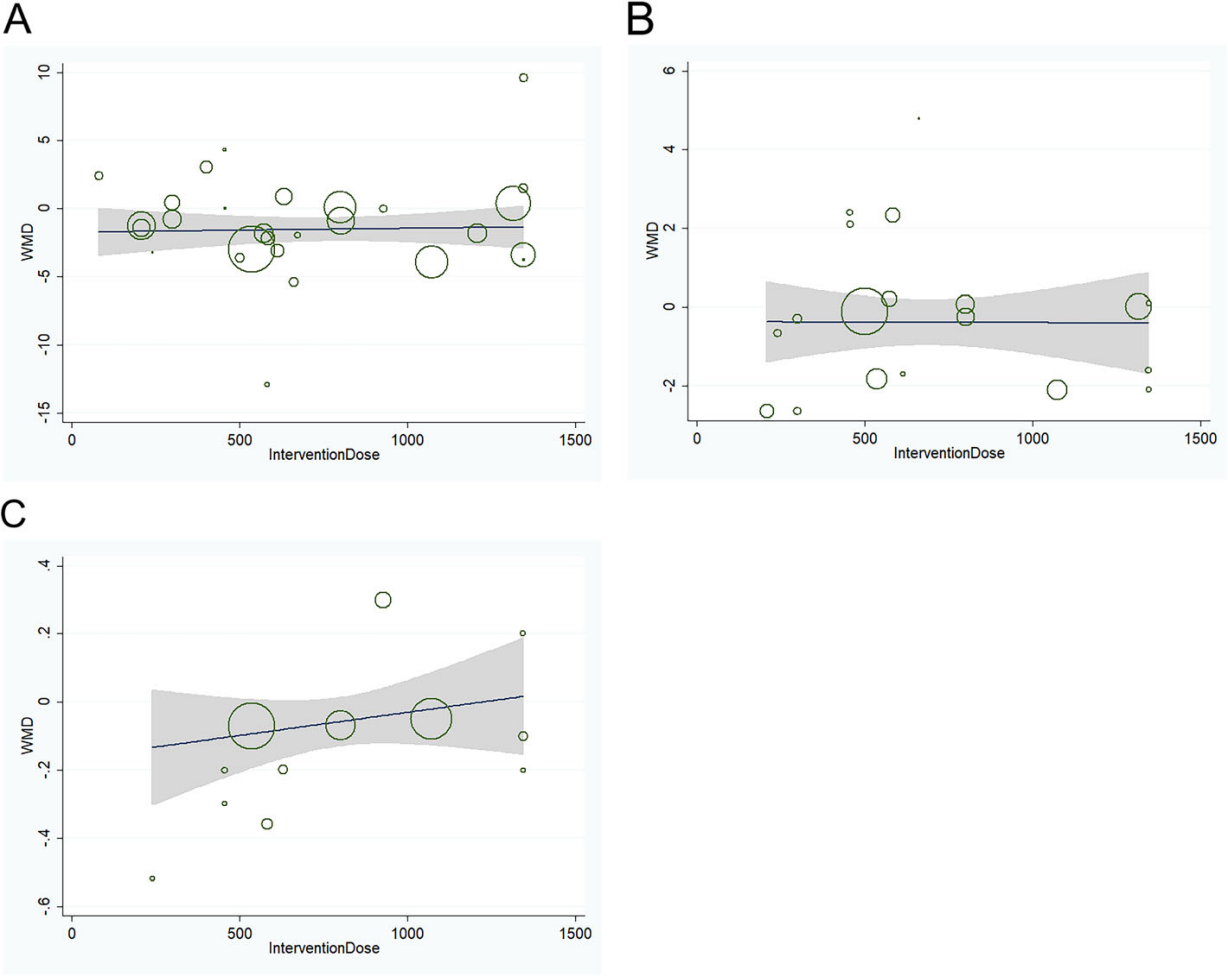
**a** Relation between the WMD of FBG and intervention dose in 27 independent randomized controlled comparisons. **b** Relation between the WMD of FBI and intervention dose in 18 independent randomized controlled comparisons. **c** Relation between the WMD of HbA_lc_ and intervention dose in 11 independent randomized controlled comparisons. Each circle represents a study, telescoped by its weight in the analysis. Meta-regression found no linear relations between WMD in FBG (*P* = 0.89), FBI (*P* = 0.97), or HbA_lc_ (*P* = 0.25) and intervention dose

Meta-regression found no linear relations between WMD in FBG, FBI or HbA_1C_ and intervention dose (Fig. 5). Furthermore, meta-regression found no linear relations between WMD in FBG or FBI and treatment duration, caffeine content, different ethnicity, intervention type and study design. In contrast, meta-regression by intervention type (beverage or capsule) did impact the WMD in HbA_1C_ for green tea consumption versus control group (*P* = 0.021), while, there was no linear relations between WMD in HbA_1C_ and other subgroups.

### Publication bias

The funnel plots of the studies were symmetrical for fasting glucose, fasting insulin, and HbA_1c_ (Supplementary Figure 1). Furthermore, the results of the Egger’s test did not support the existence of publication bias for fasting glucose (*P* = 0.18), fasting insulin (*P* = 0.58), and HbA_1c_ (*P* = 0.45).

A sensitivity analysis was performed to confirm the robustness of our findings. In a sensitivity analysis, in which one study was removed at a time and the remaining studies analyzed, the pooled reductions in fasting glucose ranged from − 1.55 mg/dL (95% CI: − 2.39, − 0.70 mg/dL) to − 1.16 mg/dL (95% CI: − 2.10, − 0.22 mg/dL); and the pooled reductions in fasting insulin ranged from − 0.59 (95% CI: − 1.21, 0.02) to − 0.23 (95% CI: − 0.87, 0.42). The result was consistent after removing each trial for both fasting glucose and fasting insulin. In the sensitivity analysis of HbA_1c_, the exclusion of one trial [22] (Basu 2011) resulted in significant reductions of-0.08 (95% CI: − 0.14, − 0.01) in HbA_1c_. However, there was no significant reduction in HbA_1c_ after the removal of other trials.

## Discussion

This meta-analysis involving 27 RCTs with 2194 subjects evaluated the effect of green tea supplementation on glycemic control. We found that green tea supplementation significantly reduced FBG concentration, while the effect of green tea on other glycemic variables such as FBI, HbA_1c_, and HOMA-IR was not significant.

Our results are consistent with some previous meta-analysis [49, 50], which also showed that green tea consumption resulted in a significant reduction in FBG. While, another previous meta-analysis [8] suggested that green tea consumption had favorable effects on decreasing both FBG and HbA_1c_ concentrations. In our study, we did not find a significant improvement in HbA_1c_ concentrations. In more than half of the included trials, the intervention duration was less than 12 weeks. However, HbA_1c_ changes need to be monitored for at least 2–3 months when evaluating the progression of diabetes. Observational prospective cohorts and case-control studies have been performed to determine the effect of green tea supplementation on glycemic control, although the results are conflicting. In particular, A large epidemiological study conducted in Japan have indicated that daily tea consumption (> 6 cups/day) was associated with a decreased risk for diabetes [51] . Some RCTs also found beneficial effects on glycemic control, including reducing fasting glucose and fasting insulin [38, 48]. In contrast, several RCTs have reported no significant correlations between green tea intake and glycemic control [24, 27]. Nonetheless, these results need to be interpreted with caution because the number of patients enrolled in most trials was too limited, at less than 100 patients; in addition, the intervention duration and catechins dosages were varied among studies. So, more RCTs with larger subjects and longer duration were needed to find out the real relationship between green tea consumption and blood glucose control.

Recent mechanistic studies have examined the effects of green tea consumption on glucose control and provided further evidence for the biological plausibility of these findings. Green tea may affect glucose control through different mechanisms. First, tea catechins have been reported to reduce carbohydrate absorption from the intestine via inhibition of intestinal sucrose, alpha-amylase, and alpha-glucosidase [10]. Second, Tea catechins might also inhibit the hepatic gluconeogenesis through regulation of the expression of gluconeogenic genes and protein-tyrosine phosphorylation in the mouse liver [52]. Third, tea catechins could enhance insulin sensitivity and glucose metabolism there by helping to prevent the development of T2DM [53]. Furthermore, Tea catechins are also powerful antioxidants that can ameliorate oxidative stress [54].

In this meta-analysis, subgroup analyses were performed based on predefined variables to identify potential sources of heterogeneity. Green tea consumption significantly decreased FBG and FBI only in subjects using green tea capsule. In addition, meta-regression also pointed out that green tea capsule was associated with HbA_1C_. Nowadays, there was still insufficient evidence on whether green tea capsule was more biologically active compared to green tea beverage in vivo or vitro studies. In addition, subgroup analyses revealed that green tea with caffeine had a more pronounced effect on FBG and HbA_1C_ than the decaffeination subgroup. As tea naturally contains caffeine in addition to catechins and other compounds, whether caffeine intake influences the glucose control of tea remains controversial [55, 56]. As there were a limited number of subjects in the subgroup analysis, these results may not be generalized.

Our study had several strengths. First, we only selected RCTs in this meta-analysis, which ensured a relatively high-quality and provided reliable inference about causality. Second, both parallel and crossover studies were included in this meta-analysis. Crossover trials are generally considered to have a more-robust design than parallel trials because of reduced intraparticipant variability. We considered it important to include all these studies because they represented a comprehensive evidence for our analysis. Third, results were less likely to be influenced by publication bias. Furthermore, subgroup analyses were undertaken to detect potential sources of heterogeneity for primary outcomes.

Our study also had several limitations. First, the studies had relatively short durations of follow-up ranging from 3 weeks to 12 months. The intervention durations were less than 12 weeks in almost half of the included studies. In particular, HbA_1c_ changes need to be monitored for at least 2–3 months when evaluating the progression of diabetes. HbA_1c_ is an important indicator for glucose control, including greater pre-analytical stability, greater convenience, and less day-to-day perturbations. In addition, it also takes a number of months to detect delayed effects of green tea on insulin resistance. Therefore, RCTs with at least 3 months intervention duration might be more appropriate to assess the effects of green tea on glycemic control. Second, although significant effect of green tea intake on fasting glucose was observed in our study, we did not provide an optimal dosage of green tea supplementation that would maximize the improvement of glycemic control as the catechin dosage varied from 80 to 1344 mg/d and no consensus has been achieved. In addition, we could not ascertain the safety margin in this meta-analysis because no serious side effects were reported in the included trials. However, mild side effects such as mild skin rashes, gastric disturbances, and abdominal bloating were reported in some clinical studies [57]. Third, the size of these trials, which ranged between 25 and 240 participants, were indeed limited. Therefore, our meta-analysis may have been underpowered to detect a true effect. Forth, the quality of RCTs included in this meta-analysis varied. Some of the RCTs did not provide detailed randomization process. Of the 27 trials, almost half of the trials were of high risk of bias, which may also affect the reliability of our findings.

## Conclusion

In conclusion, green tea intake had a favorable effect on fasting blood glucose concentration. However, green tea intake did not significantly affect fasting blood insulin or HbA_1c_. In future, high-quality larger RCTs with long-term follow-up are needed to investigate the effect of green tea supplementation on glycemic control, especially the long-term effects on fasting insulin and HbA_1c_.

## Supplementary information

**Additional file 1: Figure 1.** A. Funnel plot of green tea supplementation and FBG. B. Funnel plot of green tea supplementation and FBI. C. Funnel plot of green tea supplementation and HbA_lc_.

## Data Availability

All data generated or analyzed during this study are included in this published article.

## Abbreviations

CIs: Confidence intervals
EGCG: Epigallocatechin gallate
FBG: Fasting blood glucose
FBI: Fasting blood insulin
HbA1c: Glycated hemoglobin
HOMA-IR: Homeostatic model assessment of insulin resistance
PRISMA: Systematic Reviews and Meta-Analyses
RCTs: Randomized placebo-controlled trials
SD: Standard deviation
SE: Standard error
T2DM: Type 2 diabetes mellitus
WMD: Weighted mean difference
