# Supplementary figures and images for: Effects of green tea consumption on glycemic control: a systematic review and meta-analysis of randomized controlled trials
Source: Nutr Metab (Lond). 2020 Jul 10;17:56. doi: 10.1186/s12986-020-00469-5 (PMC7350188; doi:10.1186/s12986-020-00469-5)

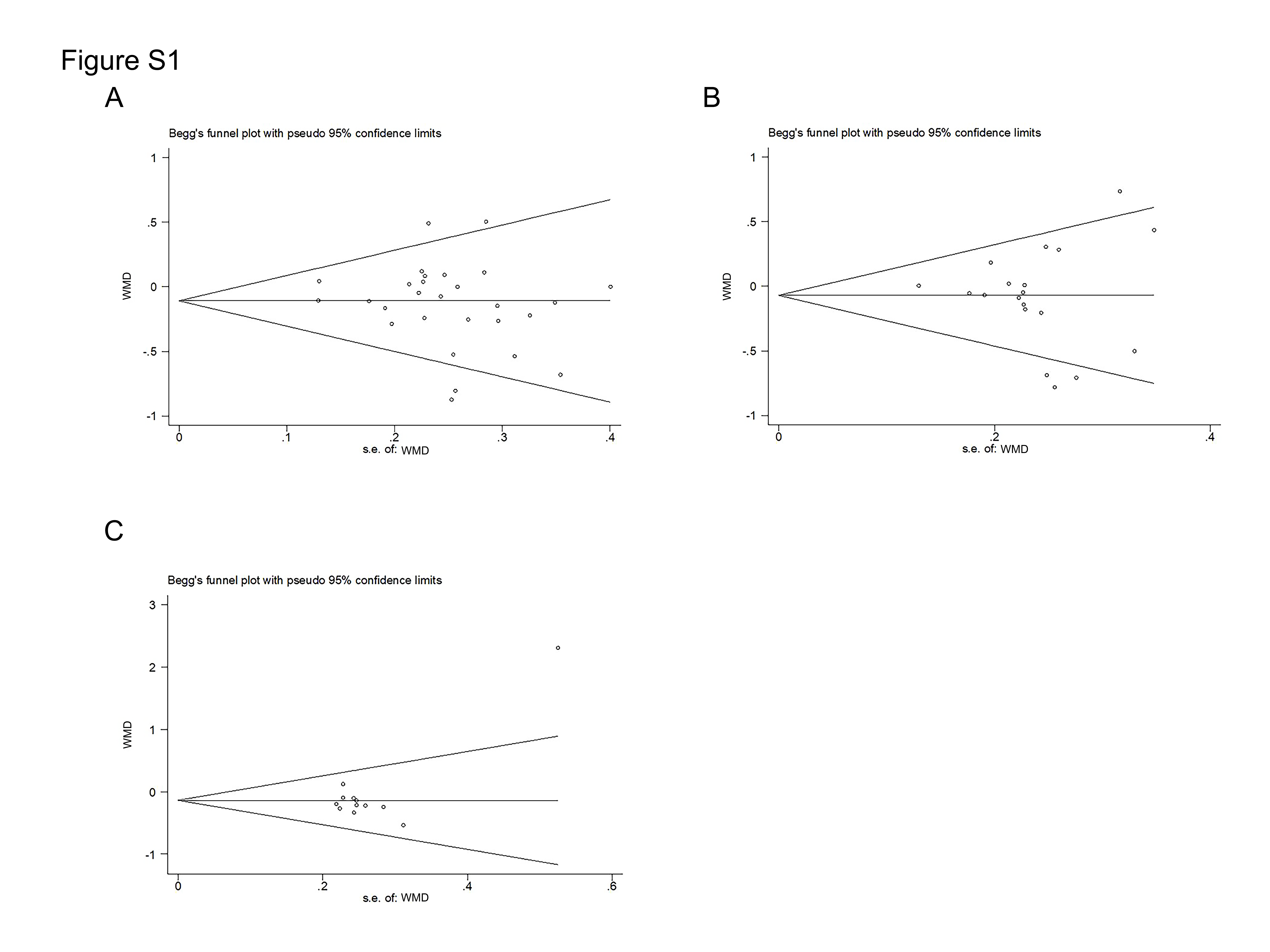

Supplement: Supplementary file 1 — Additional file 1: Figure 1. A. Funnel plot of green tea supplementation and FBG. B. Funnel plot of green tea supplementation and FBI. C. Funnel plot of green tea supplementation and HbAlc. [file 12986_2020_469_MOESM1_ESM.tif]
